# Identification of genomic regions affecting production traits in pigs divergently selected for feed efficiency

**DOI:** 10.1186/s12711-021-00642-1

**Published:** 2021-06-14

**Authors:** Emilie Delpuech, Amir Aliakbari, Yann Labrune, Katia Fève, Yvon Billon, Hélène Gilbert, Juliette Riquet

**Affiliations:** 1 GenPhySE, Université de Toulouse, INRAE, ENVT, 31320 Castanet-Tolosan, France; 2 GenESI, INRAE, 17700 Surgères, France

## Abstract

**Background:**

Feed efficiency is a major driver of the sustainability of pig production systems. Understanding the biological mechanisms that underlie these agronomic traits is an important issue for environment questions and farms' economy. This study aimed at identifying genomic regions that affect residual feed intake (RFI) and other production traits in two pig lines divergently selected for RFI during nine generations (LRFI, low RFI; HRFI, high RFI).

**Results:**

We built a whole dataset of 570,447 single nucleotide polymorphisms (SNPs) in 2426 pigs with records for 24 production traits after both imputation and prediction of genotypes using pedigree information. Genome-wide association studies (GWAS) were performed including both lines (global-GWAS) or each line independently (LRFI-GWAS and HRFI-GWAS). Forty-five chromosomal regions were detected in the global-GWAS, whereas 28 and 42 regions were detected in the HRFI-GWAS and LRFI-GWAS, respectively. Among these 45 regions, only 13 were shared between at least two analyses, and only one was common between the three GWAS but it affects different traits. Among the five quantitative trait loci (QTL) detected for RFI, two were close to QTL for meat quality traits and two pinpointed novel genomic regions that harbor candidate genes involved in cell proliferation and differentiation processes of gastrointestinal tissues or in lipid metabolism-related signaling pathways. In most cases, different QTL regions were detected between the three designs, which suggests a strong impact of the dataset structure on the detection power and could be due to the changes in allelic frequencies during the establishment of lines.

**Conclusions:**

In addition to efficiently detecting known and new QTL regions for feed efficiency, the combination of GWAS carried out per line or simultaneously using all individuals highlighted chromosomal regions that affect production traits and presented significant changes in allelic frequencies across generations. Further analyses are needed to estimate whether these regions correspond to traces of selection or result from genetic drift.

**Supplementary Information:**

The online version contains supplementary material available at 10.1186/s12711-021-00642-1.

## Background

Feed efficiency is a major driver of the sustainability of pig production systems. It represents from 50 to 83% of production costs depending on countries and systems [1]. Feed efficiency is also a major lever to reduce the environmental footprints of production [2]. In pig production, the cost of feeding is usually measured by computing the feed conversion ratio (FCR). Indeed, FCR is a ratio between two traits of interest in most breeding schemes (feed intake and growth rate), and its incorporation in selection indexes makes it difficult to accurately anticipate responses to selection on this trait and the correlated traits [3]. In 1963, Koch et al. [4] proposed residual feed intake (RFI) as an alternative to quantify feed efficiency and overcome the limits of FCR. RFI is the difference between individual feed intakes and predicted feed intake for the animals’ maintenance and production requirements. It is generally computed as a multiple linear regression of daily feed intake on production traits (growth rate and body composition traits in growing animals), and on the average metabolic body weight of the animal during the growth period, as an indicator of maintenance requirements. As a result, selection for RFI generates limited correlated responses on the other production traits, as shown in several selection experiments in pigs [5, 6], and other species [7]. However, recording accurately individual feed intake for pigs raised in groups is costly, and large efforts are devoted to facilitate the improvement of feed efficiency, by either identifying biomarkers [8, 9] or genomic markers (for instance [10, 11]). In spite of these efforts, the difficulty to identify quantitative trait loci (QTL) or genomic variants that affect feed efficiency related traits is illustrated by the PigQTLDB statistics [12]: only 394 QTL are listed for feed conversion traits, and 350 for feed intake traits, whereas more than 2000 are reported for growth traits, and more than 3200 for fatness traits (PigQTLDB, accessed September 2020, https://www.animalgenome.org/cgi-bin/QTLdb/SS/index). Genomic information acquired from established divergent lines for the trait of interest can be used to increase the power of detection of genomic variants for lowly heritable or highly polygenic traits, such as RFI in pigs [10] and litter traits in rabbits [13].

In this study, our aim was to identify genomic regions that affect RFI and other production traits in two pig lines that have been divergently selected for RFI during nine generations [5], by combining extensive genotyping of all breeding animals of the lines, and extensive phenotyping of their progeny. GWAS were applied to growth, feed intake and feed efficiency, carcass composition and meat quality traits on the full dataset. Different subsets of the population were used to be able to suggest biological hypotheses regarding the genetic background of the traits in the two divergent lines, and to decipher whether the chromosomal regions that affecting RFI differ between lines.

## Methods

### Ethic statement

All pigs were reared in compliance with national regulations and according to procedures approved by the French Veterinary Services at INRAE experimental facilities. The care and use of pigs were performed following the guidelines edited by the French Ministries of High Education, Research and Innovation, and of Agriculture and Food (http://ethique.ipbs.fr/sdv/charteexpeanimale.pdf).

### Design

The data were obtained from a divergent selection experiment on RFI carried out at the INRAE experimental unit GenESI since 2000 (Surgères, France, https://doi.org/10.15454/1.5572415481185847E12), on growing pigs from the French Large-White (LW) population. Selection procedures were previously described by Gilbert et al. [5]. In brief, the lines were established from 30 matings of LW animals (F0). From these litters, 116 males were tested to select the six most efficient (LRFI) and six least efficient (HRFI) males as founders of two divergent lines, and about 40 pairs of sibs were randomly assigned to each line. In the following generations, from G1 to G9, 96 males from each line were tested for RFI to select six extreme low or high boars depending on the line. In addition, 35 to 40 females were randomly chosen within-line in each generation to produce the next generation. No selection was applied for females. After nine generations of selection, an average inbreeding of 19% was estimated in the lines. From G1, matings were organized for at least two successive litters. Until G5, the first litter provided boar candidates for selection and future breeding females, and castrated males and females from the second parity were tested to evaluate the direct and correlated responses to selection on major production traits, including carcass composition and meat quality traits. In generation G9, the responses to selection reached − 165 g/day (LRFI line–HRFI line) for RFI (3.84 genetic standard deviations (σ_g_)), and − 270 g/day for DFI (2.11 σ_g_) (Table 1). After G5, selection was applied to parity 4 or 5, and responses to selection were measured on pigs born in parities 2 and 3. Hereafter, the breeding animals are called “breeders” and animals tested for responses to selection are called “response animals”.

**Table 1:** Number of QTL identified for each trait with the three groups of association studies

Trait	h^2^	Genetic differences in G9 (σ_g_)	Global	HRFI	LRFI	Total
DFI	0.41	2.11	2	1	3	6
ADG	0.5	0.15	1	*3*		4
FCR	0.42	2.46	2		2	4
RFI	0.13	3.84	3		2	5
carcBFT	0.4	0.037	4	*1*	*4*	9
a*_GM	0.29	0.38	2		1	3
a*_GS	0.26	0.12		4	4	8
b*_GM	0.24	0.09	1		1	2
b*_GS	0.32	1.14	6	*4*	*1*	11
L*_GM	0.2	0.38	1	*5*		6
L*_GS	0.33	2.12	2	3	4	9
pH24h_AD	0.41	1.39	2		*3*	5
pH24h_GS	0.39	1.98	4	1	1	6
pH24h_LM	0.32	1.45	4	3	4	11
pH24h_SM	0.38	1.74	3	1	1	5
WHC	0.04	0.68	3	*5*		8
MQI	0.33	1.92	4	1	1	6
LMCcalc	0.59	1.31	3		1	4
DP	0.36	0.93	3	*1*	*6*	10
Belly_W	0.28	1.90			2	2
BF_W	0.43	0.9	2	1	3	6
Ham_W	0.51	0.97	2	1	1	4
Loin_W	0.54	1.69	2		1	3
Shoulder_W	0.38	1.11		1	1	2
Total			56	36	47	139

Association studies on the full population (global-GWAS, *Global*) and for each line separately (HRFI-GWAS, *HRFI* and LRFI-GWAS, *LRFI*) were performed. Traits with more than three different QTL between the HRFI-GWAS and LRFI-GWAS analyses are indicated in italic characters. For each trait h^2^ = heritability, and responses to selection expressed in genetic standard deviations of the trait are reported as computed by Gilbert et al. [27]

DFI: daily feed intake; ADG: average daily gain; FCR: feed conversion ratio; RFI: residual feed intake; *carcBFT*: backfat thickness measured on carcass; a*_GM: a* measured on the *gluteus medius* muscle; a*_GS: a* measured on the *gluteus superficialis* muscle; b*_GM: b* measured on the *gluteus medius* muscle; b*_GS: b* measured on the *gluteus superficialis* muscle; L*_GM: L* measured on the *gluteus medius* muscle; L*_GS: L* measured on the *gluteus superficialis* muscle; *pH24h_AD*: pH 24 h after slaughter measured on the adductor femoris muscle; pH24h_GS: pH 24 h after slaughter measured on the *gluteus superficialis* muscle; pH24h_LM: pH 24 h after slaughter measured on the *longissimus dorsi* muscle; pH24h_SM: pH 24 h after slaughter measured on the *semimembranosus* muscle; WHC: water holding capacity of the *gluteus superficialis* muscle; MQI: meat quality index; LMCcalc: lean meat content of the carcass; DP: carcass dressing percentage; Belly_W: belly weight; BF_W: backfat weight; Ham_W: ham weight; Loin_W: loin weight; Shoulder_W: shoulder weight

### Phenotypes

For this study, 2426 phenotyped response animals were used, which corresponds to about 48 females and 48 castrated males per line in each generation G1 to G5, plus 700 response animals per line distributed in generations G6 to G9. All animals were raised during the growing-finishing period (~ 28 kg to ~ 107 kg) in the same growing-finishing unit comprising four rooms of four pens, each equipped with a single-place electronic feeder (ACEMA 64; Skiold Acemo, Pontivy, France). Each animal had records for body weight (BW0 at the start of the test and BW1 before slaughter) and daily feed intake (DFI) to compute average daily gain (ADG) and feed conversion ratio (FCR) during the test period. The dressing percentage (DP) was computed based on weight records of warm carcass at slaughter. Twenty four hours after slaughter, backfat thickness measured on carcass (carcBFT), and the weights of ham (Ham_W), loin (Loin_W), belly (Belly_W), shoulder (Shoulder_W), and backfat (BF_W), following a standardized cut, were recorded on the cold half carcass. The lean meat content (LMCcalc) was estimated from a linear combination of the weights of carcass ham, loin, and backfat, expressed as a percentage of the half-carcass weight [14]: LMC (%) = 25.08 − 1.23 backfat (%) + 0.87 loin (%) + 0.73 ham (%). Meat quality measurements included pH on the *adductor femoris* (AD), *semimembranosus* (SM), *gluteus superficialis* (GS), and *longissimus dorsi* muscles (LM), colorimetry L*, a* and b* on GS and *gluteus medius* muscle (GM), and water-holding capacity (WHC) assessed on GS according to the procedure described by Charpentier et al. [15]. Finally, a meat quality index (MQI) was calculated from measurements of the pH on SM, L* on GS and WHC according to the model proposed by Tribout et al. [16]. RFI was defined as the residual of a multiple linear regression as follows: RFI = DFI − (1.48 × ADG) + (23.2 × LMCcalc) −  (99.1 × AMBW), where AMBW is the average metabolic body weight during the test period and is equal to (BW1^1.6^ − BW0^1.6^)/[1.6 (BW1 − BW0)] [17]. Contemporary group (group of around 45 animals born in the same week and contemporarily tested in a given room), gender and pen size were added as fixed effects in the model, as described by Gilbert et al. [5].

### Genotyping

Genomic DNA was purified from individual biological samples of the sires and dams of all generations using standard protocols. Over the time of the study, two different Illumina medium-density SNP chips were used according to the genotyping protocols defined by the supplier (Technological Center, Genomics and Transcriptomics Platform, CRCT Toulouse). A first genotyping batch comprising 286 animals (12 sires from each generation G0 to G6, and G0, G3 and G6 dams) was genotyped for 64,232 SNPs using the Porcine SNP60v2 BeadChip (60K SNPs chip), and a second batch of 1356 animals (complementary breeding animals of the generations G0 to G6 and sires and dams of the following generations) was genotyped using the Porcine HD Array GGP chip comprising 68,516 SNPs (70K SNPs chip). Genotypes were obtained using the Genome Studio software (V2.0.4) and coded as 0, 1 and 2 corresponding, respectively, to individuals homozygous for the minor allele, heterozygous and homozygous for the major allele. In addition, 32 G0 founders equally distributed between the lines (12 G0 sires, and 20 G0 dams that contributed most to the subsequent generations based on pedigree information) were genotyped with the Affymetrix Axiom Porcine HD Genotyping Array chip (Gentyane Platform, UMR 1095 INRAE Clermont-Ferrand) consisting of 658,692 SNPs (650K SNPs chip).

For each SNP panel, quality control was performed using the PLINK software (V1.90) [18]: SNPs with a call frequency (CF) lower than 95% and a minor allele frequency (MAF) lower than 1% were excluded, and animals with a call rate (CR) lower than 90% were discarded. Deviations from Hardy*–*Weinberg equilibrium were also assessed with a *p*-value of 10^–10^. Unmapped SNPs and SNPs located on the sex chromosomes were removed based on the Sscrofa11.1 assembly of the reference genome (https://www.ensembl.org/Sus_scrofa/Info/Index) [19].

### Imputation of genotypes

Two successive imputations were performed using the FImpute software [20]. A first level of imputation was performed with markers on the 60K and 70K SNPs chips, based on 29,957 common SNPs, to homogenize the medium-density genotyping data available for the 1632 breeders of the lines. This leads to an intermediate dataset of 66,988 SNPs that are imputed from both medium-density (MD) chips (60K and 70K SNPs chips). In a second step, the genotypes of the high-density (HD) SNPs chip were imputed for all breeders using the HD SNP genotypes of the 32 G0 founders. A set of common 45,708 SNPs was available between the MD imputed genotypes and the HD SNP chip. Finally, 570,447 SNPs distributed along the 18 pig autosomes were available for 1632 breeding animals.

To evaluate imputation accuracy, first, five successive batches of 1000 SNPs were randomly selected among the common SNPs between the 60 and 70K SNP chips. For each SNP batch, the genotypes of these SNPs were set as missing for all animals genotyped with the 60K SNPs chip and imputed from the 70K SNPs chip information. Therefore, 5000 SNPs with real and imputed genotypes were used to compute Pearson’s correlations for each of the 286 pigs with 60K genotypes. Similarly, five batches of 1000 SNPs were randomly selected among the common SNPs between both MD SNP chips, animals genotyped with the 70K SNPs array were re-coded as missing, and Pearson’s correlations between true and imputed genotypes were computed for the 1346 animals with 70K SNP genotypes. Then, to evaluate the imputation quality to the HD level, the same strategy of removing successively five batches of 1000 SNPs from the data was applied using SNPs that were in common among the three chips. In addition, a leave-one-out approach was applied to the 32 individuals with HD genotypes to evaluate the imputation accuracy.

In addition, a multi-dimensional scaling (MDS) analysis was performed using the *cmdscale()* function in the R software (V.3.6.2, R Core Team 2019) based on a identity-by-state matrix constructed with the PLINK software [18].

### Predicted genotypes in response animals

Response animals did not have genotypes themselves. An average expected genotype was computed for each animal from the imputed 650K genotypes of their parents. For each SNP, each individual was given the average genotype of the parents (0, 0.5, 1, 1.5 or 2), thus within a litter, all animals were assigned the same genotypes. Thus, depending on the class of genotypes, the obtained genotype represented an approximation of the real genotype: (i) genotypes 0 and 2 were certain, as they resulted from two homozygous parents for the same allele (0 × 0 0 and 2 × 2 2), (ii) genotypes 0.5 and 1.5 included combinations of a homozygous genotype for one allele and a heterozygous genotype (0 × 1 0 or 1 and 1 × 2 1 or 2), and (iii) genotype 1 was the most heterogeneous class, with a mixture of true genotypes (0 × 2 1) and uncertain genotypes (1 × 1 0 or 1 or 2). Animals whose parents had a missing genotype were excluded from the analysis.

### Genome-wide association studies

GWAS analyses were performed using the GEMMA software (version 0.97) [21] on response animals with their own phenotypes and their average genotypes from the parents. Phenotypes were adjusted for significant fixed effects and covariates (pen size, herd, sex, and contemporary groups for in vivo measurements, slaughter date as fixed effects, and slaughter age as covariate for traits recorded at the abattoir, and slaughter BW as covariate for carcBFT) using linear models as proposed in Aliakbari et al. [22]. The resulting residues were integrated as phenotypes in GEMMA. To account for the structure of the population in the GWAS analyses, a pedigree relationship matrix $\mathbf{A}$ was computed. Association analyses were performed on the 24 traits available for the 2426 response animals.

The statistical model used to test one marker at a time was $\mathbf{y}=\mathbf{x}\beta +\mathbf{Z}\mathbf{u}+{\varvec{\upvarepsilon}}$, where $\mathbf{y}$ is the vector of adjusted phenotypes for all individuals; $\mathbf{x}$ is a vector of genotypes at the tested marker; $\beta$ is the effect of the tested marker; $\mathbf{u}$ is a vector of random additive genetic effects distributed according to $N(0, \mathbf{A}\lambda {\tau }^{-1})$, with $\lambda$ the ratio of the additive genetic variance and the residual variance ${\tau }^{-1}$ and $\mathbf{Z}$ the incidence matrix (identity matrix in this case); ${\varvec{\upvarepsilon}}$ is a vector of residuals $N(0, \mathbf{I}{\tau }^{-1})$, with $\mathbf{I}$ the identity matrix. In GEMMA, an efficient exact algorithm is implemented to first estimate $\lambda$, and next derive $\widehat{\beta }$ and $\widehat{\tau }$ for each marker [23].

Three types of populations were considered for GWAS. First, the full dataset, which combines the two lines, was analyzed in a global analysis (thereafter called global-GWAS). Then, to evaluate if some QTL were segregating in one line only, the analyses were repeated within line (thereafter called lines-GWAS, or HRFI-GWAS and LRFI-GWAS when only one line was referred to).

For each analysis, the distributions of the test statistics of the GWAS of each trait were checked using quantile–quantile plots (Q-Q plot), and we computed the regression coefficients of the observed to the expected distribution under H_0_. Inflation factors were on average 1.17 ± 0.15 for all analyses, indicating low deviations from the distribution of the test statistic under H_0_. However, a correction factor was applied to all the analyses to control type-I errors, by dividing each chi square statistic by the corresponding inflation factor, following the genomic control approach proposed by Devlin and Roeder [24]. The test nominal *p*-values were computed according to this new chi square statistic.

To account for the multiple testing issue in the computation of genome-wide type-I errors, the significance threshold was obtained after a Bonferroni correction as follows:

$$-\text{log}10\left(\frac{0.05}{\sum_{i=1}^{nb chr}{number \,of\, independent\, tests}_{i})}\right),$$

where the number of independent tests was computed as the sum of the number of independent tests for each chromosome. For each chromosome, this number was the number of principal components required to describe 99.6% of the genotype variability, obtained from a principal component analysis applied to the correlation matrix between genotypes of the SNPs on the considered chromosome (square root (r^2^) of linkage disequilibrium (LD) between each pair of SNPs, Gao et al. [25]). The resulting genome-wide threshold (4.5 corresponding to 1690 independent tests) was used to select significant associations for each type of analysis. In addition, a cut-off of 3 (chromosome-wide threshold) was used only to assess whether a significant region identified in one analysis was suggestive in another one.

To define QTL intervals, the genome was divided into 1-Mb windows following the Sscrofa11.1 assembly of the reference genome. First, for each analysis (HRFI-GWAS, LRFI-GWAS and global-GWAS performed for each trait), the 1-Mb windows with at least one SNP with a significant *p*-value at 5% genome-wide (−log_10_(*p*-value) ≥ 4.5) were retained, and adjacent windows with significant signals were combined into a single "QTL-window" per trait. In a second step, all the QTL windows were combined across traits using the same approach as above: adjacent and overlapping QTL-windows were fused, thus allowing the definition of a complete list of "QTL-regions". When a QTL-region was significant for several traits, for each one, the most significant marker and the associated allelic substitution effect was retained to tag the QTL (trait × region) for this trait in further analyses – thereafter called SNP-QTL.

The QTL positions were compared to previously mapped QTL in pigs using the pigQTLdb database [12], and QTL significant for RFI trait were screened for functional candidate genes using the Ensembl annotation V.101 (August 2020).

### Changes in allelic frequencies of SNP-QTL

The power of detection in GWAS is strongly influenced by the allelic frequencies of the analyzed markers [26]. Within each QTL region, the different SNP-QTL were considered to examine the changes in allele frequencies with line selection. It should be noted that in addition to selection, changes in allele frequencies can also be due to genetic drift, especially in small closed populations. For instance, under the Wright-Fisher model (panmixia, no selection, N = 40) in our lines, genetic drift would result in generation 9 in standard deviations of allele frequencies of 0.164 for SNPs with an initial frequency of 0.5. However, our objective was not to test if allele frequencies responded to selection, but to illustrate changes in allele frequencies with time, accounting for all generations, in QTL regions. These SNP-QTL allele frequencies were estimated for the response animal genotypes, i.e. from their average genotypes. To investigate how selection affected allele frequencies, and thus power of detection, allele frequencies were computed by adding animals from one generation at a time, starting from G1 individuals only. Then, the allele frequencies by adding G2 response animals were obtained by combining genotypes of G1 and G2 response animals, and so on until G9. The estimated frequencies in G9 (using all the animals from G1 to G9) corresponded to the informativeness of the markers used in the main lines-GWAS. In each line, a regression of the generation number (1 to 9) on the SNP allele frequencies was then applied to test changes in allelic frequencies on cumulative datasets across generations. For each SNP-QTL, the significance of the slope was tested in each line using a Wald test. The comparison of the slopes (the regression coefficients of the allelic frequencies) between lines highlighted four distinct cases: (i) markers with frequencies that did not change with line selection (no slope differed from zero with the Wald tests), (ii) markers co-selected in the two lines (slopes differed from zero and had identical signs), (iii) markers selected in opposite directions in the lines (slopes differed from zero with different signs), and (iv) markers with frequencies that changed in one line only (slope different from zero in one line only). Using only the significant slope values, a QTL evolution score was computed for each SNP-QTL as (9 generations * (|slope _HRFI_| +|slope _LRFI_|)) and to summarize their evolution per trait, an average score over all SNP-QTL for each trait was computed.

## Results

### Quality control and imputation of genotypes

True SNP genotyping data were available for all sires and dams from G0 to G9. The quality control of the genotypes was carried out first for each SNP chip independently. With a CR threshold of 90%, 10 animals genotyped with the 70k SNP chip and no individual genotyped with the 60K and 650K SNP chips were discarded (see Additional file 1: Table S1). For the SNPs, 15,114 SNPs from the 60K SNP chip (5776 for CF < 95% and 9125 for MAF < 1%), 11,891 SNPs from the 70K SNP chip (5323 for CF < 95% and 6568 for MAF < 1%), and 99,587 SNPs from the HD SNP chip (53,735 for CF < 95% and 45,852 for MAF < 1%) were removed. No SNP was removed with the Hardy–Weinberg equilibrium filter. In total, genotypes of 286 animals for 49,118 SNPs from the 60k SNP chip, genotypes for 1346 animals for 56,625 SNPs from the 70K SNP chip, and finally genotypes for 32 animals for 559,105 SNPs from the HD SNP chip were retained for further analyses (see Additional file 2: Table S2).

To obtain HD genotypes for all parents in the design, two successive runs of imputations were performed. First, the imputation of the missing genotypes on each MD support (60K and 70K SNP chips) allowed us to obtain genotypes for 66,988 SNPs for all sires and dams. The imputation accuracy was on average 0.995 regardless of the generation of the imputed individuals (see Additional file 3: Figures S1a and 1b). A second run of imputation was applied to all breeding animals from the 32 founder individuals genotyped with the HD SNP chip. The imputation accuracy was also high, with average accuracies around 0.979 (see Additional file 3: Figure S1c). A few animals in G0 and G3 had accuracies lower than 0.97. The accuracy estimated via the leave-one-out approach confirmed the values estimated with the correlations, with an average of 0.975 (see Additional file 3: Figure S1d). In total, genotypes for 570,447 SNPs were obtained for all parents from G0 to G9.

**Fig. 1:**
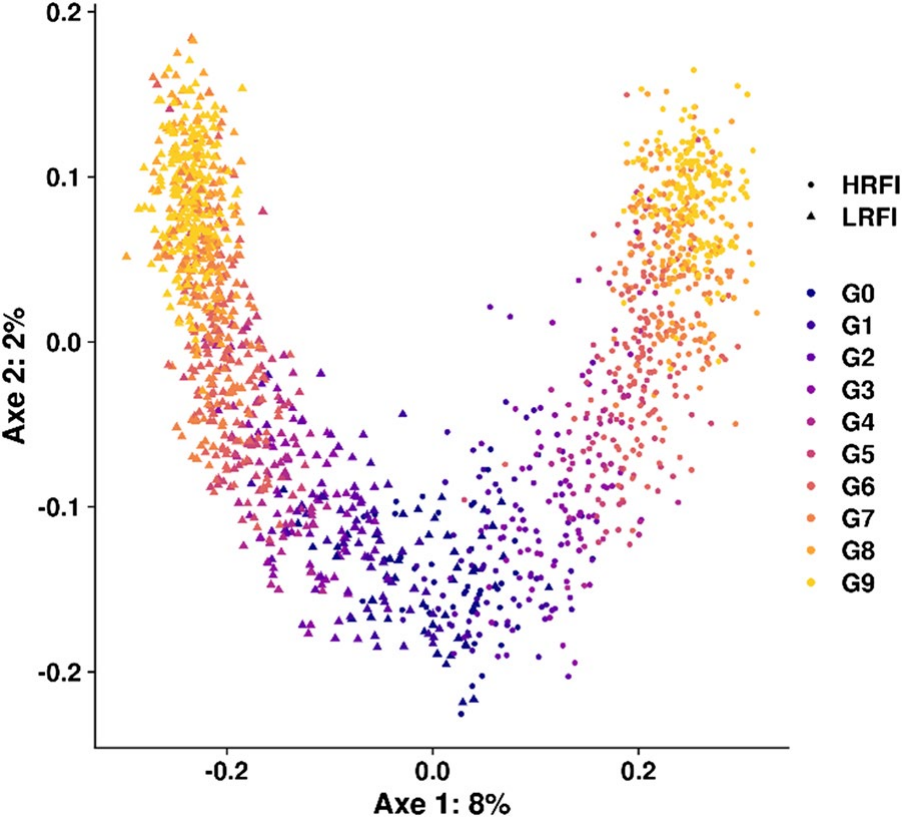
The first two axes of the multidimensional scaling (MDS) analysis, based on the 570,447 genotypes. Points represent individuals (corresponding to all sires and dams of the population, N = 1632) and colors are generations

An MDS analysis was performed on the genotype matrix to represent the changes in genomic content of the lines with generations (Fig. 1). The first component corresponded to the dispersion of individuals according to the lines, and the second component corresponded to the successive generations in both lines.

### Genome-wide association studies

From the imputed genotypes of all parents, an average genotype was computed for all response animals. Thus, genotypes coded 0, 0.5, 1, 1.5 or 2 were available for 2426 individuals. In total, the design included 596 full-sib families including 4.07 (± 2.9) individuals on average. Within a sibling, all individuals shared the same average genotype. The proportions of the five possible genotypes were estimated for each SNP and each individual in the design, with indication of their uncertainty. For each SNP, the proportion of certain genotypes, corresponding to classes 0, 1 (half of them) and 2, represented 1276 genotypes on average, i.e. 53% of the individuals, with a median of 1130 genotypes that are certain, this proportion being higher for SNPs with an extreme MAF. In addition, for each individual, among the 66,988 SNPs for the MD imputed genotypes considered in the calculation, from 31,132 to 40,852 SNPs (an average of 35,232 SNPs) were predicted with certainty (see Additional file 4: Figure S2).

**Fig. 2:**
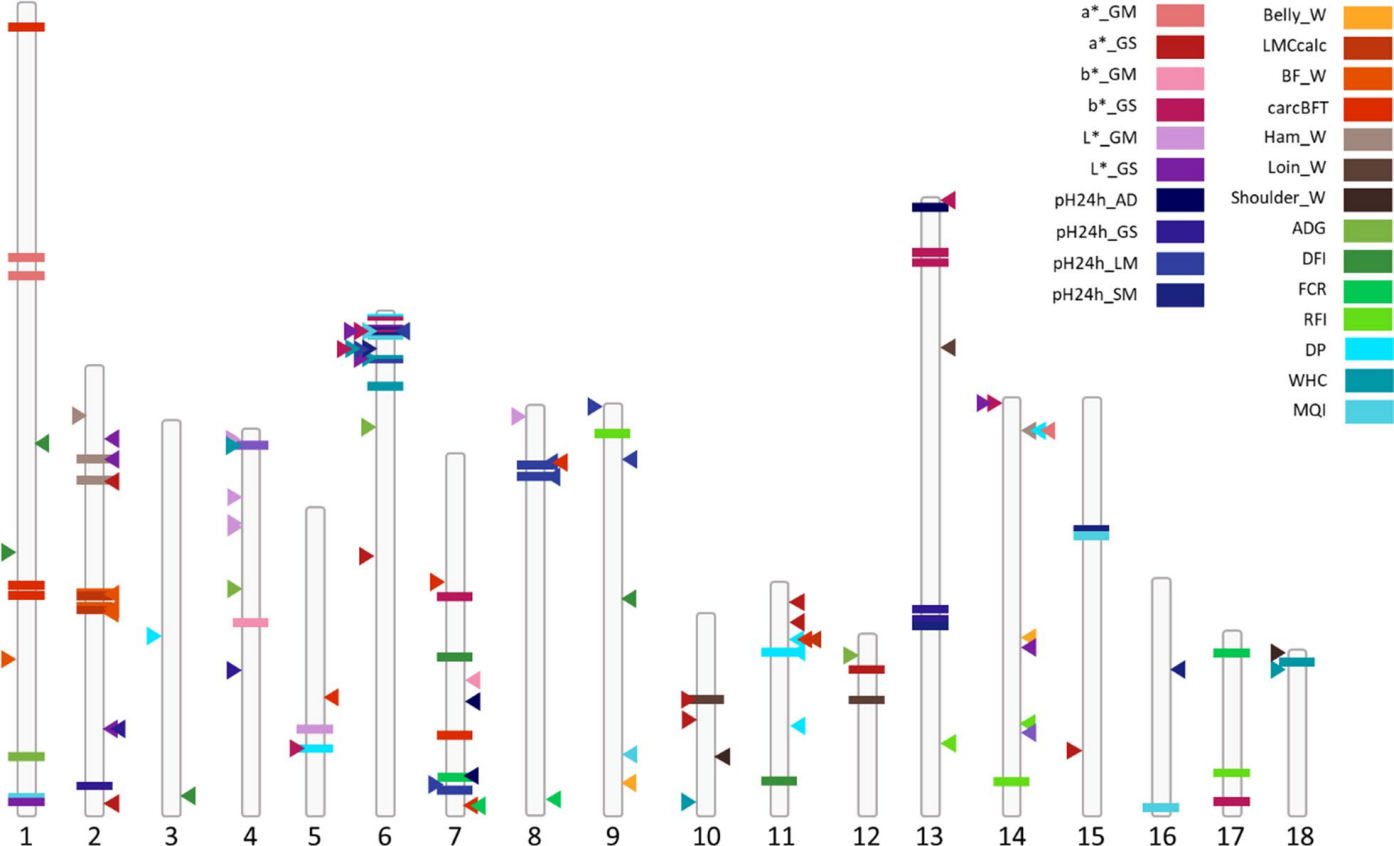
Location of all SNP-QTL identified on the 18 autosomes from the Global-GWAS, LRFI-GWAS and HRFI-GWAS. The SNP-QTL corresponding to Global-GWAS are represented by horizontal bars, LRFI-GWAS by arrows to the right of the chromosomes and HRFI-GWAS by arrows to the left of the chromosomes. Each color represents one of the 24 traits; LRFI: low RFI line; HRFI: high RFI line; DFI: daily feed intake; ADG: average daily gain; FCR: feed conversion ratio; RFI: residual feed intake; carcBFT: backfat thickness measured on carcass; a*_GM: a* measured on the *gluteus medius* muscle; a*_GS: a* measured on the *gluteus superficialis* muscle; b*_GM: b* measured on the *gluteus medius* muscle; b*_GS: b* measured on the *gluteus superficialis* muscle; L*_GM: L* measured on the *gluteus medius* muscle; L*_GS: L* measured on the *gluteus superficialis* muscle; pH24h_AD: pH 24 h after slaughter measured on the adductor femoris muscle; pH24h_GS: pH 24 h after slaughter measured on the *gluteus superficialis* muscle; pH24h_LM: pH 24 h after slaughter measured on the *longissimus dorsi* muscle; pH24h_SM: pH 24 h after slaughter measured on the *semimembranosus* muscle; WHC: water holding capacity of the *gluteus superficialis* muscle; MQI: meat quality index; LMCcalc: lean meat content of the carcass; DP: carcass dressing percentage; Belly_W: belly weight; BF_W: backfat weight; Ham_W: ham weight; Loin_W: loin weight; Shoulder_W: shoulder weight

First, association studies corresponding to global-GWAS were carried out on all response animals, for each of the 24 traits. Significant regions were selected by applying a genome-wide threshold of 4.5. Forty-five regions of 1 Mb (31 regions), 2 Mb (6 regions), 3 Mb (7 regions) or 8 Mb (1 region) were significant for at least one trait, corresponding to 56 QTL-windows (trait × region) for the global-GWAS. For all traits (except Belly_W, Shoulder_W and a*_GS), at least one QTL was detected in these analyses (Fig. 2), the list and characteristics of these QTL are reported in Additional file 5: Table S3.

**Fig. 3:**
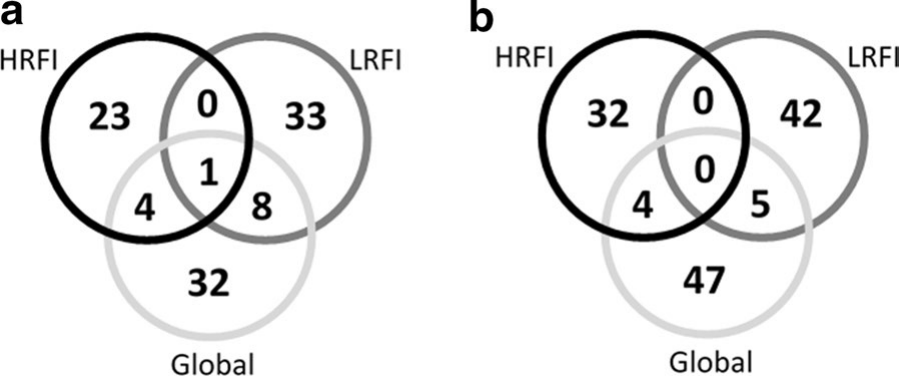
Comparison of GWAS results obtained from Global-GWAS (Global), HRFI-GWAS (HRFI) and LRFI-GWAS (LRFI). **a** Comparison of the number of identical regions and **b** comparison of the number of identical QTL (trait x region)

To assess whether the identified QTL regions were identical and shared between the two lines, complementary GWAS analyses were performed per line, using either the set of individuals from G1 to G9 of the HRFI line or the set of individuals from G1 to G9 of the LRFI line. The QTL identified with the three analyses were compared (Table 1 and Fig. 2). As an example of the outcome of these analyses, Manhattan plots for RFI obtained with the global-GWAS and lines-GWAS are reported in Additional file 6: Figure S3. For the analyses performed by line, the number of regions detected for a trait could differ between lines. For instance, more loci were detected in the HRFI line for ADG, b*_GS, L*_GM and WHC, whereas more regions were detected in the LRFI line for carcBFT, pH24h_AD and DP. In the HRFI line, 36 QTL were identified in 28 regions, and in the LRFI line, 47 QTL were identified in 42 regions. Only one region overlapped between the two lines: on SSC6, a region located between 7 to 10 Mb affected pH24h_LM in LRFI and L*_GS, b*_GS, and MQI in HRFI, which are highly correlated traits related to meat quality (Fig. 3).

Cut weights were the traits with the smallest number of QTL (1 to 3 per analysis) (Table 1). Meat quality measurements had the largest number of QTL (up to 6). Nineteen regions associated with growth, feed intake, and feed efficiency were detected, including five regions associated with RFI and four with FCR.

**Fig. 4:**
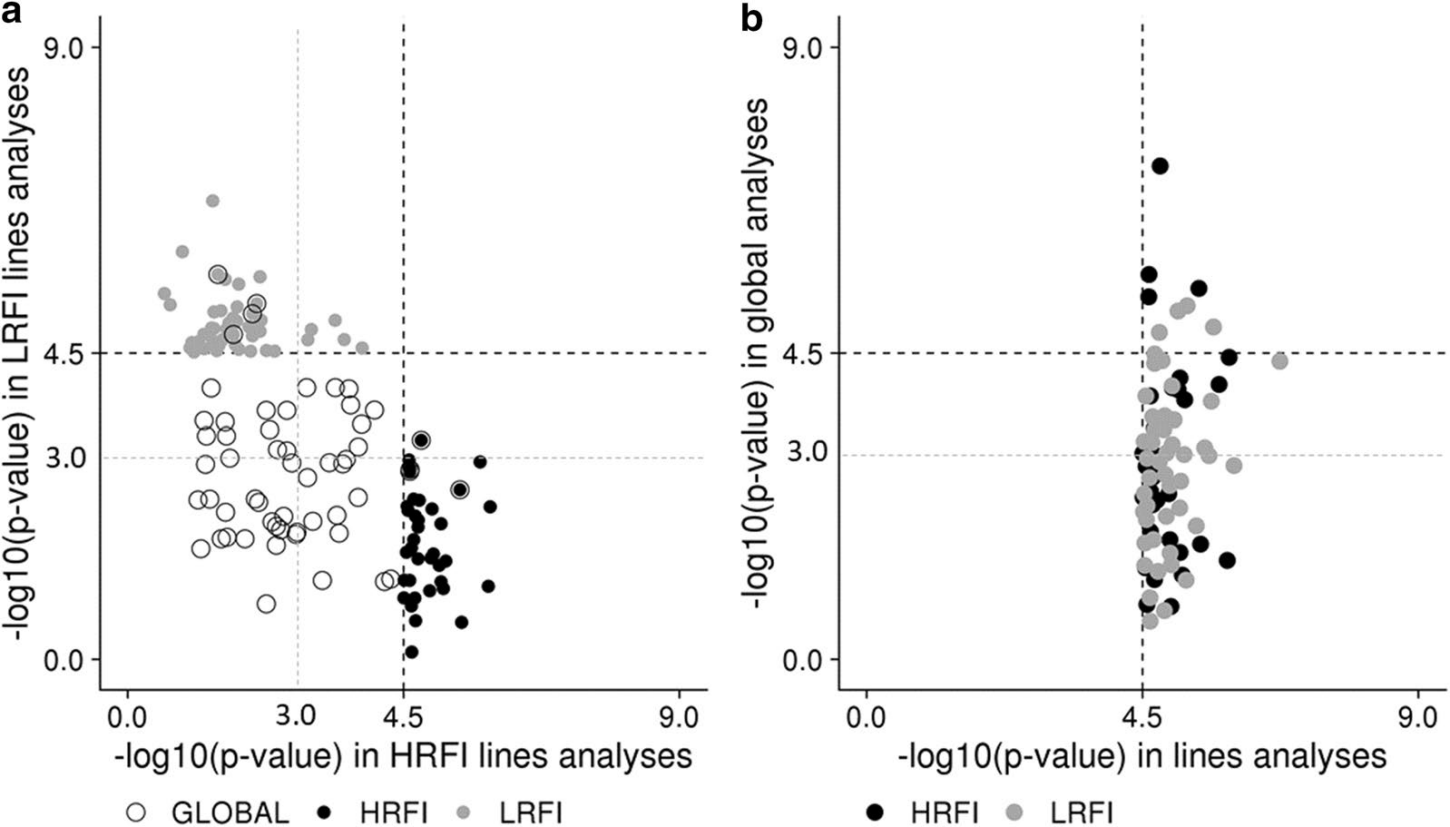
Plot of the − log_10_(*p*-value) of the SNP-QTL. The − log_10_(*p*-value) are obtained in first case with the two lines analyses for all SNP-QTL detected for the lines or the global analyses (**a**), and in second case obtained with the global analysis for SNP-QTL detected with the GWAS performed per line (**b**)

Thirteen regions were shared between the 45 regions identified in the global-GWAS and the 69 unique regions from the analyses per line, with only five common regions between the global-GWAS and HRFI-GWAS analyses, nine common regions between the global-GWAS and LRFI-GWAS, and the SSC6 region described above detected in the three analyses (Fig. 3a). Among these regions, only nine QTL (trait x region) were identified jointly in the global-GWAS and in one of the lines-GWAS (Fig. 3b), and none was shared in the three analyses. Thus, very few QTL were common between the three GWAS (Fig. 2). To assess whether a SNP-QTL significant in one analysis reached significance or suggestive thresholds in the other analyses, their *p*-values were compared. First, in the comparison between the lines-GWAS (Fig. 4a), most of the SNP-QTL detected via HRFI-GWAS had −log_10_(*p*-values) generally lower than the suggestive threshold of 3 in the LRFI-GWAS. Similar results were obtained comparing SNP-QTL of the LRFI-GWAS to their *p*-values with the HRFI-GWAS. For the SNP-QTL significant in the global-GWAS, the −log_10_(*p*-values) with the lines-GWAS were intermediate and exceeded the suggestive threshold in one of the lines for several QTL.

In addition, for the SNP-QTL corresponding to the QTL detected in the line analyses (HRFI-GWAS and LRFI-GWAS), the −log_10_(*p*-values) obtained in the global-GWAS were also low (Fig. 4b), with more than the half (56.6%) of the SNP-QTL having -log_10_(*p*-values) lower than 3.

### Change in allele frequencies across generations

**Fig. 5:**
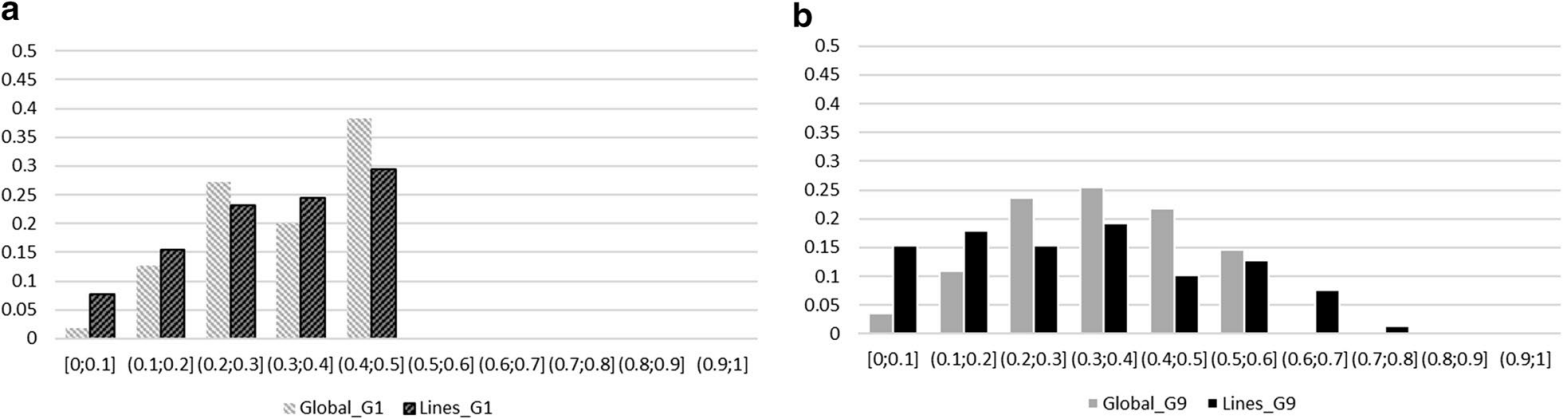
Distribution of SNP-QTL allele frequencies of Global-GWAS (in grey) and Lines-GWAS (in black). Distribution representing individuals from the line of the significant analysis **a** in G1 generation (G1 individuals only) and **b** in G9 generation (G1 to G9 individuals)

The allele frequencies of the SNP-QTL detected either in the global-GWAS or lines-GWAS were evaluated in G1 to G9 to reflect the informativeness of these GWAS (called G9 hereafter) and in G1. When the SNP-QTL was detected in the global-GWAS, all response animals were used to compute the frequencies; for SNP-QTL from the lines-GWAS, only the animals of the significant analysis (HRFI-GWAS or LRFI-GWAS) were used. The resulting frequency histograms are shown in Fig. 5. In G1 only, the distribution of the allelic frequencies of the SNP-QTL of the global-GWAS and that of the SNP-QTL of the lines-GWAS did not differ significantly (Fig. 5a). In G9, the distribution of the SNP-QTL allelic frequencies differed largely between the two types of analyses (Fig. 5b): 85.5% of the SNP-QTL of the global-GWAS remained in the same range of frequencies, between 0.2 and 0.6, whereas only 57.7% of the SNP-QTL of the lines-GWAS had allele frequencies within that range of values (P < 0.001 for a Chi^2^ with 1 df, when comparing between the two types of analyses the number of SNP-QTL with frequencies between 0.2 and 0.6 with the number of SNP-QTL with other frequencies). In addition, 9% of the SNP-QTL of the lines-GWAS had a frequency higher than 0.6, whereas no marker reached such frequencies among the SNP-QTL of the global-GWAS.

**Fig. 6:**
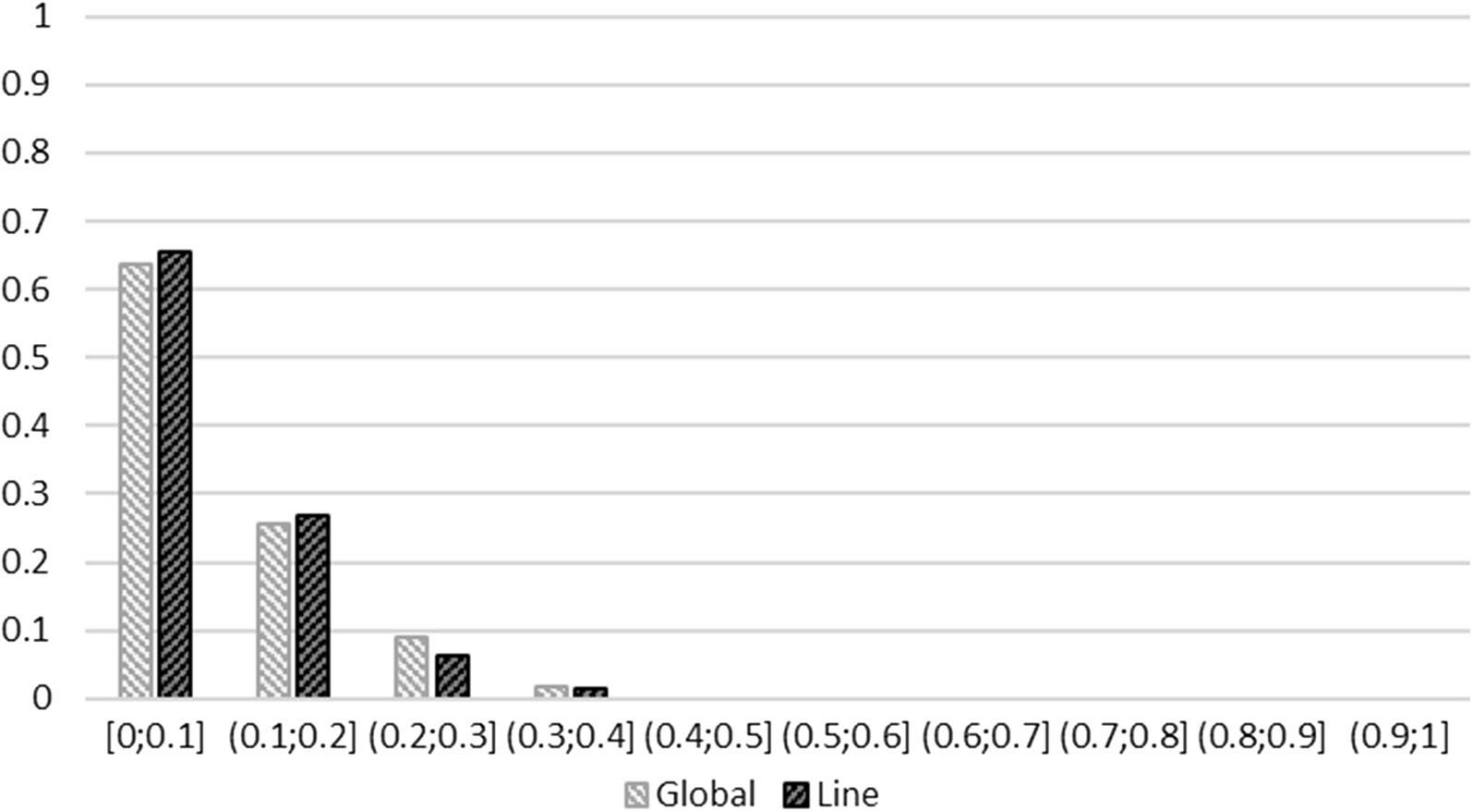
Distribution of differences in allele frequencies between the lines. The differences in allele frequencies are the absolute values between lines for SNP-QTL resulting from the Global-GWAS and Lines-GWAS in G1

**Fig. 7:**
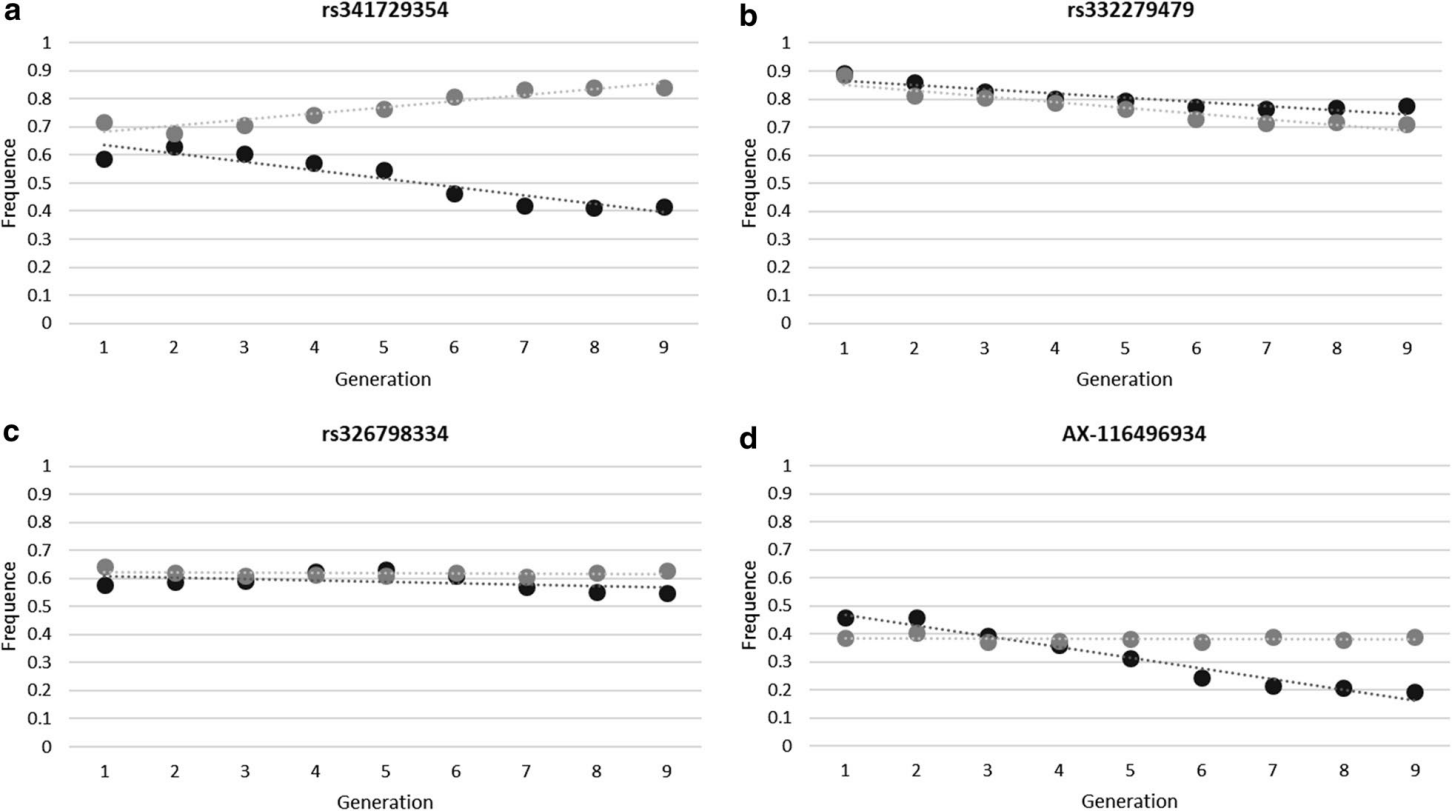
Linear regression of the generation number on the allele frequencies computed in each line. Allele frequencies were estimated in the two lines by combining, for each generation, individuals of the generation n with the previous ones (animals from generation G1 to G n-1). Allelic frequencies evolutions are reported for SNP-QTL corresponding to **a** no-evolution, **b** co-evolution in both lines, **c** opposite-evolution, and **d** evolution only in one line, situations

**Fig. 8:**
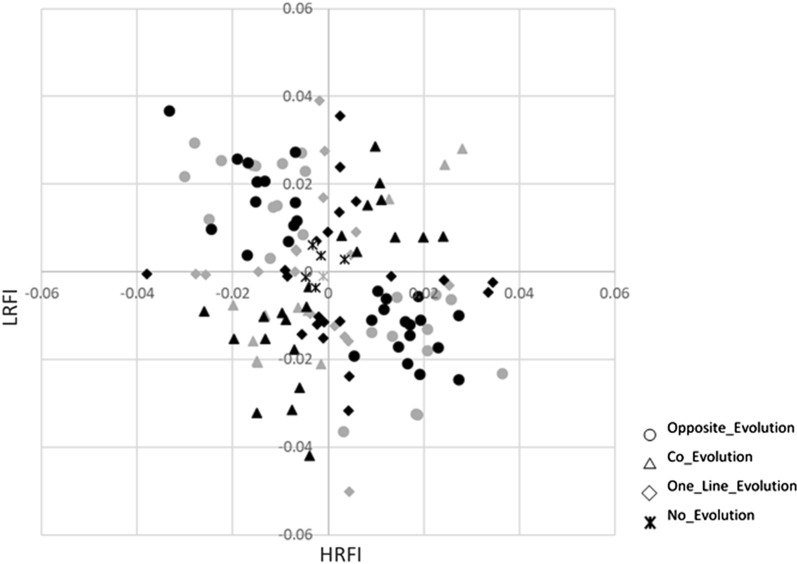
Slopes of the linear regression equations of the allele frequencies on the nine generations. Slopes were calculated in each line, for all SNP-QTL identified with Global-GWAS (in grey) and Lines-GWAS (in black). Four situations (differentiated by different labels) were identified according to the significance of the slope (different from zero with p < 0.05 with a Wald test) in one or the two lines

In addition to the estimation of the global allelic frequencies, we evaluated if in each line the detected SNP-QTL in each type of analysis evolved differently. First, the differences in allele frequency between the HRFI and LRFI lines were estimated in the G1 generation (at the beginning of the selection) (Fig. 6). Regardless of the analysis (global- or lines-GWAS) in which the SNP-QTL was detected, initially more than 63% of the SNP-QTL showed small differences in line frequency (< 0.1) and less than 11% of the SNP-QTL showed a difference in line frequency greater than 0.2. These SNP-QTL were not particularly detected in one or the other type of analysis. Next, to better describe the changes in allele frequency across generations, frequencies of SNP-QTL from the global-GWAS and lines-GWAS were successively estimated in each line by adding data from the next generation to the previous generations: G1 allele frequencies were obtained from G1 individuals only, G2 allele frequencies were obtained from G1 and G2 individuals etc. Using the nine resulting frequencies computed for each line, a linear regression of the generation number on the allele frequencies was applied within line (Fig. 7). The comparison between lines of the regression coefficients of the allelic frequencies highlighted four distinct cases (Fig. 8). Altogether, the allelic frequencies of 4.5% of the SNP-QTL did not change with selection (Fig. 7a), 24.8% of the markers were co-selected in the two lines (Fig. 7b), 41.3% evolved in opposite directions in the two lines (divergence) (Fig. 7c), and 29.3% of the markers had frequencies that changed in one line only (17.3% in LRFI and 12% in HRFI) (Fig. 7d). Again no difference in the distribution of the SNP-QTL by category was identified in either type of analysis (*p*-value = 0.51 for a Chi^2^ with 3 df).

**Fig. 9:**
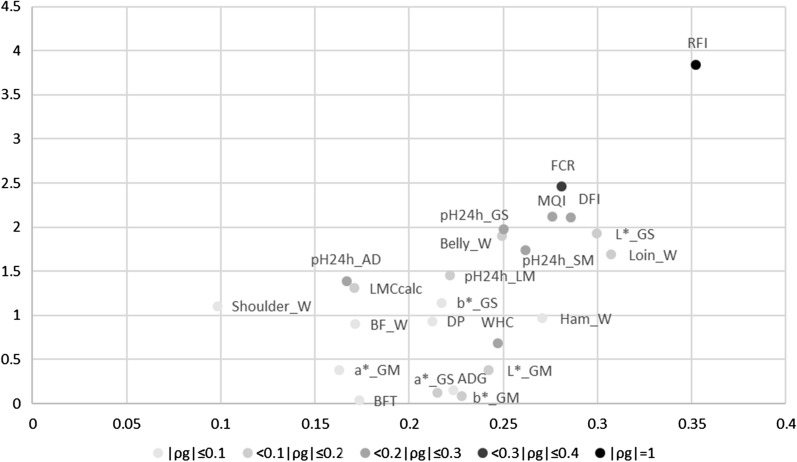
Genetic differences in G9 between the two lines. The genetic differences were expressed in genetic standard deviations of the trait (σ_g_) as a function of the average evolution of allelic frequencies in the QTL regions of the trait between the two lines. The magnitude of the genetic correlation between each trait and RFI is indicated with a grey gradient; DFI: daily feed intake; ADG: average daily gain*; *FCR: feed conversion ratio; RFI: residual feed intake; carcBFT*:* backfat thickness measured on carcass; a*_GM: a* measured on the *gluteus medius* muscle; a*_GS: a* measured on the *gluteus superficialis* muscle; b*_GM: b* measured on the *gluteus medius* muscle; b*_GS: b* measured on the *gluteus superficialis* muscle; L*_GM: L* measured on the *gluteus medius* muscle; L*_GS: L* measured on the *gluteus superficialis* muscle; pH24h_AD: pH 24 h after slaughter measured on the adductor femoris muscle; pH24h_GS: pH 24 h after slaughter measured on the *gluteus superficialis* muscle; pH24h_LM*:* pH 24 h after slaughter measured on the *longissimus dorsi* muscle; pH24h_SM: pH 24 h after slaughter measured on the *semimembranosus* muscle; WHC: water holding capacity of the *gluteus superficialis* muscle; MQI: meat quality index; LMCcalc: lean meat content of the carcass; DP: carcass dressing percentage; Belly_W: belly weight; BF_W: backfat weight; Ham_W: ham weight; Loin_W: loin weight; Shoulder_W: shoulder weight

For RFI in the two lines, four of the five detected QTL corresponded to regions that were selected in opposite directions in the lines, with strong differences in line frequencies: two RFI SNP-QTL showed differences in allelic frequency between lines greater than 0.2 in G1 and the other two RFI SNP-QTL showed large changes in allelic frequency (regression slope > 0.024/generation). To summarize the changes in SNP-QTL allele frequencies for each trait, an average evolution score between G1 and G9 was computed using the estimated evolution scores of the different SNP-QTL detected for each trait. These averages were between 0.09 (Shoulder_W) and 0.35 (RFI). A correlation coefficient of 0.63 was then estimated between the genetic line differences in G9 computed previously for the 24 different traits [27] (Table 1) and these averages (Fig. 9).

## Discussion

The objective of this study was to identify QTL that affect RFI and production traits in pig lines that have been divergently selected for RFI and to understand if the traits had different genetic backgrounds between the lines. By optimizing the genotyping to reach a sufficient power of detection of QTL in the full design and in the two lines, separately, QTL were detected for all traits and hypotheses about the trait genetic background in the two lines can be formulated.

### Using average parental genotypes to detect QTL

While the use of SNP chips now enables the genotyping of an individual at a reasonable cost, the genotyping of a design comprising several thousands of individuals still represents a significant investment. In each generation of our design, at least two parities were produced, one to select future breeders, and one to control the responses to the selection on feed consumption, growth and meat quality traits via measurements at the slaughterhouse. After nine generations of selection, around 2500 "response animals" had phenotypes. These individuals have the advantage of having individual records for unmeasured traits in breeders (post-mortem measurements). To optimize the costs, we genotyped all 1632 breeders on MD SNP chips to exhaustively survey the segregating alleles in the design. In addition, the 32 main contributors to the design were chosen from the G0 sires and dams to be genotyped using the HD SNP chip, and an imputation step was carried out to have HD genotypes for all breeding individuals. The strong pedigree relationships in the design enabled a very good quality of HD imputation, since they help to better detect long haplotypes used to infer missing SNPs [28]. A second step was carried out, so that each response non-genotyped animal could have a genotype. Such imputation of non-genotyped animals has been used in cattle [29] as part of genomic evaluations to increase the size of reference populations. In cattle, the most common situation is to determine by imputation the genotypes of the dams of the bulls, knowing the genotypes of the maternal grandsire, one (or more) offspring and the sires with which they were mated [30]. In such cases, the strategy takes advantage of family information (Mendelian rule of allele transmission) and combines it with allele frequencies and LD between markers at the population level. In our case, at each generation n, all response animals had both parents genotyped at generation n-1. Given these trio structures, an expected genotype at each position could be deduced from the genotypes of the parents using simple segregation rules: since the genotypes were coded as an allelic dosage for one reference allele, the genotype expectation for each offspring was simply the average of the genotypes of its two parents. As a result, 2426 animals with genotypes (predicted) and phenotypes were available for subsequent GWAS analyses.

### Understanding the differences in the regions detected between analyses

The regions detected with each type of analysis (global- or lines-GWAS) were very different and only 10 among the 129 detected QTL were shared between global-GWAS and lines-GWAS. The SNP-QTL detected with the global-GWAS were far from reaching the threshold of significance in the lines-GWAS. Similarly, most of the SNP-QTL detected with the lines-GWAS were far from reaching the threshold of significance in the global-GWAS. Although the number of individuals included in the global-GWAS was more than twice that in the line analyses, the addition of individuals belonging to the other line seems to have reduced the power of detection of QTL segregating in the first line. Even if the allelic frequencies of the SNP-QTL detected in the global-GWAS or lines-GWAS were comparable in G1, they largely differed after nine generations of selection, i.e. more SNPs with low allele frequencies were identified with the lines-GWAS. The pedigree kinship matrix was used in the GWAS model to correct for the strong genomic structure of the population. Although this classical approach is successful to control type-I errors of the analyses, it also limits the power of detection of QTL in highly differentiated regions between lines, since their link with trait variability would be absorbed into the additive genetic component of the model. Thus, global-GWAS essentially allow the detection of regions that segregate at intermediate frequencies in both lines. As an alternative, the analyses carried out by line allow the detection of regions that are close to fixation with selection in one of the lines. From these results, it seems that the power of detection related to allele frequencies in each line is the main difference between QTL-SNPs detected with the lines-GWAS and global-GWAS. Thus, given the power of the design, it is likely that the biological pathways involved in RFI variability in the two lines are similar, but with different contributions to the trait in each line, contrary to some previous hypotheses [10, 27].

### Comparison with published regions

Among the five QTL detected for RFI, three regions were detected close to previously published RFI QTL. The region on SSC14 at 130–131 Mb is close to the region described by Do et al. [31] who proposed *G-protein-coupled receptor kinase 5* (*GRK5*) (129,114,449–129,343,412 bp) as a candidate gene. Wang et al. [32] reported that a GRK5 deficiency led to insulin resistance and hepatic steatosis, and to decreases in diet-induced obesity and adipogenesis in mice. At the position 131,181,710–131,579,703 bp, *FGFR2* (*fibroblast growth factor receptor 2*) could also be an interesting candidate gene. All four FGF receptors and several FGF ligands are present in the intestine and are key players in controlling cell proliferation, differentiation, epithelial cell restitution, and stem cell maintenance. *FGFR2* is expressed in the human ileum and throughout adult mouse intestine [33]. The second region closest to published RFI QTL is the 184–186 Mb interval on SSC13 near the QTL reported by Bai et al. [34] and Do et al. [31]. In this region, *TMPRSS15* (*transmembrane serine protease 15*) is an interesting candidate gene. This gene encodes an intestinal enzyme that is responsible for initiating the activation of pancreatic proteolytic proenzymes. It catalyzes the conversion of trypsinogen to trypsin, which in turn activates other proenzymes including chymotrypsinogen procarboxypeptidases and proelastases. *TMPRSS15* has been associated to enterokinase deficiency, a life-threatening intestinal malabsorption disorder characterized by diarrhea and failure to thrive [35]. On SSC17, two RFI QTL have been published by Do et al. [31] close to the *SOGA1* gene (*suppressor of glucose, autophagy-associated protein 1*, 40,020,107–40,098,992 bp) and by Onteru et al. [10] close to the *DOK5* gene (*docking protein 5*, 55,391,074–55,541,561 bp). These two QTL surround the region that we detected and could correspond to a unique QTL. At position 48,090,077–48,100,816 bp and at position 48,132,911–48,149,732 bp, respectively, *PLTP* and *ZNF335* are additional candidate genes. In humans, Coleman et al. [36] identified the region encoding ZNF335 as a susceptibility locus for the coeliac disease, a chronic immune-mediated disease triggered by the ingestion of gluten [36]. The PLTP (phospholipid transfer protein) transfers phospholipids from triglyceride-rich lipoproteins to high-density lipoprotein (HDL). In addition to regulating the size of HDL particles, this protein may be involved in the metabolism of cholesterol. *PLTP-*KO mice absorb less cholesterol than wild-type mice, and also have a deficient secretion in the intestine [37].

### Potential pleiotropic effects

The large number of traits recorded in our design and the known genetic correlations between these traits [27] enable the detection of pleiotropic regions, i.e. regions that affect multiple traits. Among the four regions detected for FCR, no QTL co-localized with a RFI QTL. For the other traits, only two QTL were detected within 10 Mb of the RFI QTL: one QTL at 8 Mb influencing pH24h_LM on SSC9 between 1 and 2 Mb, and one QTL on pH24h_AD at 1 Mb of the QTL for RFI located at 113–114 Mb on SSC14. Compared to the previously published QTL regions for RFI, we identified only one QTL for DFI in the region described by Guo et al. [38] on SSC3 between 126 and 128 Mb. In spite of the reported correlations between these traits and RFI, among the 36 QTL detected in our study for DFI, MQI, WHC, pH24h_AD, pH24h_GS, and pH24h_SM, only one QTL co-located with the RFI QTL identified in our study or in previously published studies.

### Changes in QTL allele frequencies and trait responses to selection

The allele frequencies of the majority of the detected regions changed between generations G1 and G9, with more than 70% of the regions for which SNP-QTL evolved in opposite directions or in one line only. However, the magnitude of the changes in allelic frequencies of the QTL regions varied among the traits, and was strongly correlated with previously reported line differences in G9 [27]. Indeed, the regions with the largest changes in allelic frequencies were detected for RFI, which was the trait used for selection. For the other traits, the higher the genetic correlation with RFI, the higher the variation in allelic frequency of the associated QTL regions. As a result, QTL that affect FCR, DFI and MQI showed the largest changes in allelic frequency with generations. The responses of QTL that affect meat quality traits are consistent with the high and early responses to selection previously detected in this experimental population for these traits [5]. Altogether, our analyses underline a clear relationship between the quantitative responses to selection of the traits and the changes in allelic frequencies in some QTL regions, which potentially point out to chromosomal regions that were selected during the experiment. Nevertheless, it is important to note that changes in allelic frequencies can also result from genetic drift. In such populations with a small effective size and strong directional selection, the power of detection of signatures of selection using standard methodologies [39] can be low due to the major effect of genetic drift on the changes in allele frequencies. However, recently-developed new methods, based on genetic time series could provide new insights for the detection of regions under selection in small populations [40].

## Conclusions

In this study, our aim was to characterize the molecular architecture of RFI in two lines that have been divergently selected for this trait. In addition to efficiently detecting known and new QTL regions, the combination of GWAS performed per line or simultaneously using all individuals allowed the identification of candidate regions on the genome and to understand how the genomes of both lines have evolved. Analyzing the allelic frequencies from G1 to G9, we identified that most of the differences in the results of QTL detection between the global or the two lines-GWAS were due to differences in informativity of the SNP-QTL in the two lines after nine generations of selection. Even if we cannot distinguish whether these evolutions in allelic frequencies are a direct effect of the directional selection or are due to drift, the regions detected can explain the responses to selection of different traits reported before. In addition, we conclude that the majority of the QTL regions followed divergent patterns in the lines, and that the same metabolic pathways were certainly involved in both lines. We identified several new regions that underlie RFI variability and propose new candidate genes that complement the data acquired in previously published analyses.

## Supplementary Information

**Additional file 1: Table S1.** Number of animals used for the analyses after quality control. Details of the number of animals before and after application of a filter on the call rate (CR) were given for chips (60K, 70K and 650K SNPs chips), imputation levels (MD/HD imputation) and average genotypes calculated from the genotypes of both parents (HD predicted).

**Additional file 2: Table S2.** Number of SNPs used for the analyses after quality control. Details of the number of SNPs before and after application of filters on the call frequency (CF) and the frequency of minor allele (MAF) were given for chips (60K, 70K and 650K SNPs chips), imputation levels (MD imputation and HD imputation) and average genotypes calculated from the genotypes of both parents (HD predicted).

**Additional file 3: Figure S1.** Correlations between true and imputed genotypes for animals genotyped on 60K, 70K or 650K SNPs chip. For each analysis, correlations were estimated setting 5000 SNPs as missing (5 batches of 1000 SNPs) on one chip among SNPs in common between the two arrays used. Animals are sorted and colored by generation. Correlations between true and imputed genotypes (a) for the 286 animals genotyped with the 60K SNPs chip using animals with 70K genotypes as reference population, and (b) for the 1346 animals genotyped with the 70K SNPs chip using animals with 60K genotypes as reference. (c) Correlations between true and imputed genotypes after imputation to 650K SNPs from the imputed medium density genotypes. (d) Correlations between true and imputed genotypes based on the leave-one-out cross-validation.

**Additional file 4: Figure S2.** Proportion of certain expected genotypes per animal, per SNP and in relation to the MAF of the SNPs. The proportion of certain genotypes corresponds to expected genotypes from parents which are homozygous for the same allele or homozygous for opposite alleles, and half of the genotypes from matings of two heterozygous parents were also taken into account. This proportion was studied per individual for the 66,988 SNPs of the 60K SNPs chip (a), per SNP for the 2426 pigs (b) and finally per SNP while taking into account the MAF of each SNP (c).

**Additional file 5: Table S3.** QTL regions detected with the three groups of association studies. These QTL regions were found from the full population (Global-GWAS) and from each line separately (HRFI-GWAS and LRFI-GWAS). DFI: daily feed intake; ADG: average daily gain; FCR: feed conversion ratio; RFI: residual feed intake; carcBFT: backfat thickness measured on carcass; a*_GM: a* measured on the *gluteus medius* muscle; a*_GS: a* measured on the *gluteus superficialis* muscle; b*_GM: b* measured on the *gluteus medius* muscle; b*_GS: b* measured on the *gluteus superficialis* muscle; L*_GM: L* measured on the *gluteus medius* muscle; L*_GS: L* measured on the *gluteus superficialis* muscle; pH24h_AD: pH 24 h after slaughter measured on the adductor femoris muscle; pH24h_GS: pH 24 h after slaughter measured on the *gluteus superficialis* muscle; pH24h_LM: pH 24 h after slaughter measured on the *longissimus dorsi* muscle; pH24h_SM: pH 24 h after slaughter measured on the *semimembranosus* muscle; WHC: water holding capacity of the *gluteus superficialis* muscle; MQI: meat quality index; LMCcalc: lean meat content of the carcass; DP: carcass dressing percentage; Belly_W: belly weight; BF_W: backfat weight; Ham_W: ham weight; Loin_W: loin weight; Shoulder_W: shoulder weight

**Additional file 6: Figure S3.** Manhattan plots for GWAS of RFI trait in global, HRFI line or LRFI line populations. The plot shows the − log10(p-values) for all SNPs in the analysis against their genomic position. Changes in color represent different chromosomes. The dashed line represents the threshold for genome wide significance (threshold of -log10(*p*-value) = 4.5).

## Data Availability

The datasets used and/or analysed during the current study are available from the corresponding author on reasonable request.
