# Supplementary figures and images for: Identification of genomic regions affecting production traits in pigs divergently selected for feed efficiency
Source: Genet Sel Evol. 2021 Jun 14;53:49. doi: 10.1186/s12711-021-00642-1 (PMC8201702; doi:10.1186/s12711-021-00642-1)

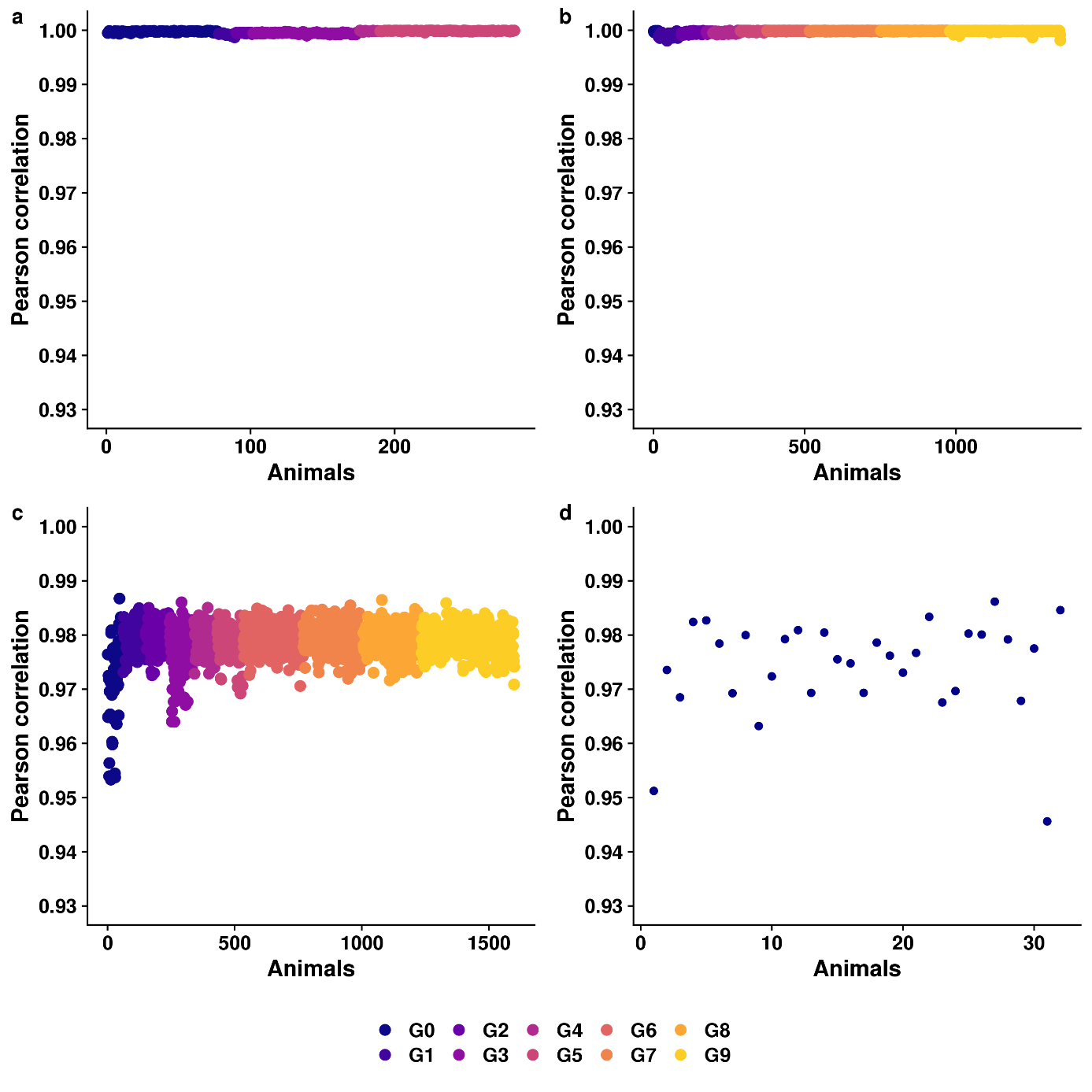


Additionnal file 3

Supplement: Supplementary file 3 — Additional file 3: Figure S1. Correlations between true and imputed genotypes for animals genotyped on 60K, 70K or 650K SNPs chip. For each analysis, correlations were estimated setting 5000 SNPs as missing (5 batches of 1000 SNPs) on one chip among SNPs in common between the two arrays used. Animals are sorted and colored by generation. Correlations between true and imputed genotypes (a) for the 286 animals genotyped with the 60K SNPs chip using animals with 70K genotypes as reference population, and (b) for the 1346 animals genotyped with the 70K SNPs chip using animals with 60K genotypes as reference. (c) Correlations between true and imputed genotypes after imputation to 650K SNPs from the imputed medium density genotypes. (d) Correlations between true and imputed genotypes based on the leave-one-out cross-validation. [file 12711_2021_642_MOESM3_ESM.docx]

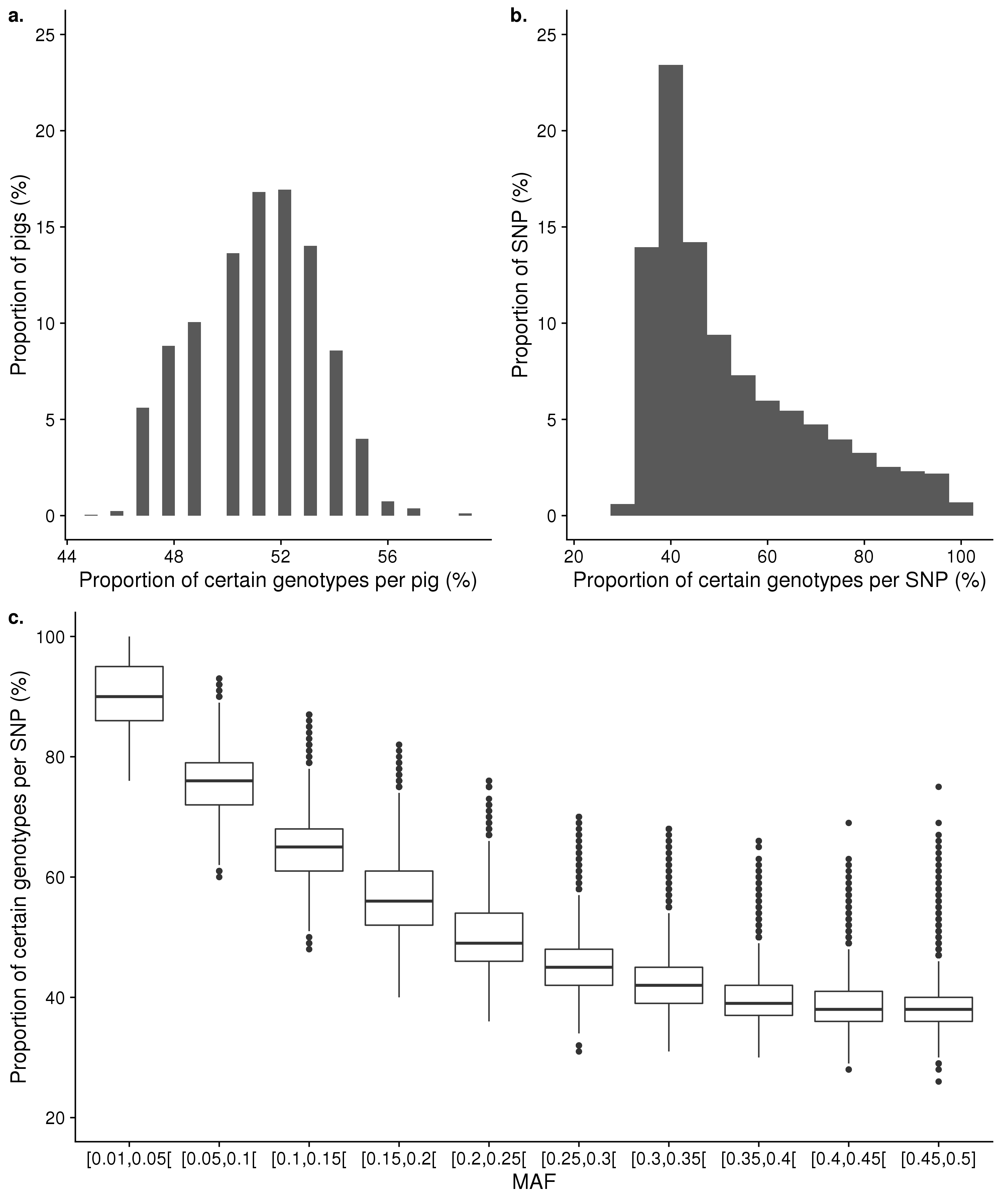


Additional file 4

Supplement: Supplementary file 4 — Additional file 4: Figure S2. Proportion of certain expected genotypes per animal, per SNP and in relation to the MAF of the SNPs. The proportion of certain genotypes corresponds to expected genotypes from parents which are homozygous for the same allele or homozygous for opposite alleles, and half of the genotypes from matings of two heterozygous parents were also taken into account. This proportion was studied per individual for the 66,988 SNPs of the 60K SNPs chip (a), per SNP for the 2426 pigs (b) and finally per SNP while taking into account the MAF of each SNP (c). [file 12711_2021_642_MOESM4_ESM.docx]

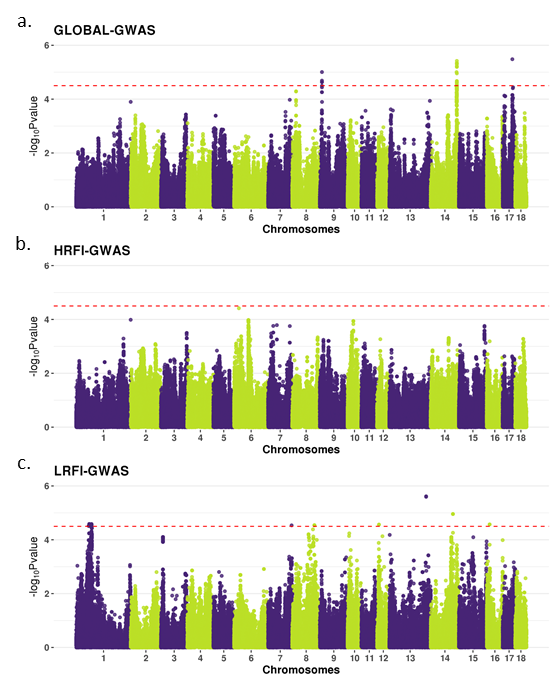


Additional file 6

Supplement: Supplementary file 6 — Additional file 6: Figure S3. Manhattan plots for GWAS of RFI trait in global, HRFI line or LRFI line populations. The plot shows the − log10(p-values) for all SNPs in the analysis against their genomic position. Changes in color represent different chromosomes. The dashed line represents the threshold for genome wide significance (threshold of -log10(p-value) = 4.5). [file 12711_2021_642_MOESM6_ESM.docx]
